# Improving the Quality of Care Coordination for Children and Young People with Intellectual Disability/Developmental Disorder in the Emergency Department Post CPD-QI Intervention (Motivated for Change Program)

**DOI:** 10.3390/children13020255

**Published:** 2026-02-12

**Authors:** Yu-Na Kim, Natalie Ong, Gail Tomsic, Ruth Bowron, Jacqueline Milne, Abbie Lucien, Karl Pobre, Shefali Jani

**Affiliations:** 1 The Children’s Hospital at Westmead, Corner Hawkesbury Road and Hainsworth Street, Westmead, NSW 2145, Australia; 2 Child Development Unit, The Children’s Hospital at Westmead, Corner Hawkesbury Road and Hainsworth Street, Westmead, NSW 2145, Australia; natalie.ong@health.nsw.gov.au (N.O.); gail.tomsic@health.nsw.gov.au (G.T.); ruth.bowron@health.nsw.gov.au (R.B.); jacquelinemilne@bigpond.com (J.M.); 3 Faculty of Medicine and Health, University of Sydney, Camperdown, NSW 2050, Australia; karl.pobre@health.nsw.gov.au (K.P.); shefali.jani@health.nsw.gov.au (S.J.); 4 Faculty of Medicine and Health, University of New South Wales, Sydney, NSW 2033, Australia; a.lucien@unsw.edu.au; 5 Department of Emergency Medicine, The Children’s Hospital at Westmead, Corner Hawkesbury Road and Hainsworth Street, Westmead, NSW 2145, Australia

**Keywords:** motivated for change program, children and young people, intellectual disability, developmental disorder, care coordination, emergency department

## Abstract

**Highlights:**

**What are the main findings?**

- Care coordination elements were identified in emergency department care for children and young people with ID/DD.
- Care coordination practices were more evident and intentional after implementation of the Motivated for Change training program.

**What are the implications of the main findings?**

- Disability-focused training and practical system-level tools (e.g., the Top 5 Tile) can strengthen care coordination in paediatric emergency settings.
- Embedding reasonable adjustments and integrating child life therapists or dedicated liaison roles support child-centred communication and family partnerships for children and young people with ID/DD.

**Abstract:**

**Background/Objectives:** Children and young people with intellectual disability/developmental disorder (ID/DD) face inequities in hospital care, including poor communication, limited reasonable adjustments, and fragmented coordination. This study examined the presence of care coordination elements within staff and caregiver experiences and explored how these practices were influenced by a locally delivered staff training program implemented in a tertiary paediatric emergency department (ED) in New South Wales, Australia (Motivated for Change). **Methods**: A qualitative pre–post design was used, incorporating staff and caregiver interviews and ED observations to evaluate the program. This study included 22 observations (10 baseline, 12 post-intervention) and 15 interviews (six baseline, nine post-intervention) with staff and caregivers. The intervention included three one-hour training sessions and practical tools such as the digital Top 5 Tile This study represents a secondary use of existing data, applying a previously established care coordination framework and its associated definitions. Data were analysed using the framework method by five members of the research team. **Results:** Post-intervention, staff more consistently engaged parents and caregivers, made tailored adjustments, and used the Top 5 Tile to support information continuity. Child life therapists played a pivotal role in advocating for families and modelling inclusive practices. The findings mapped strongly to the framework domains of communication, proactive care planning, and aligning resources to needs, though systemic constraints remained. **Conclusions:** Targeted training and structured tools can strengthen care coordination for children and young people with ID/DD in EDs, improving safety and quality of care. Broader implementation across other departments and evaluation of sustainability are warranted.

## 1. Introduction

People with intellectual disability/developmental disorder (ID/DD) experience disproportionate disadvantage across multiple domains, including economic, employment and social participation, contributing to significantly poorer health outcomes [1,2]. They face higher rates of comorbidities, reduced access to preventive healthcare, increased hospitalisation, and premature mortality [3,4]. Due to their complex health needs, adults with ID/DD are more likely to experience frequent hospital admissions, longer stays, and greater reliance on carers or hospital staff for support [1,5,6].

ID/DD, emerging during the developmental period, is defined by notable limitations in intellectual and adaptive functioning across conceptual, social, and practical domains [7]. The global prevalence is estimated to be approximately 1%, although this figure varies depending on the population studied and the methods used to define and measure ID/DD [8,9,10]. In Australia, around 286,000 people live with ID/DD, representing approximately 1.1% of the total population. This accounts for 6.5% of all individuals who report having a disability [11]. Among children aged 0–14 years, the prevalence is higher, with 4.5% living with ID/DD [12].

Children and young people with ID/DD face similarly stark health inequities, including a high prevalence of chronic health conditions and barriers to participation in community and educational settings, often compounded by emotional and behavioural difficulties [1,13]. In hospital environments, they face heightened risk due to medication errors, communication breakdowns with families and between healthcare teams, poor health literacy, and limited capacity for self-advocacy by the young person and their families. These factors contribute to misdiagnosis and suboptimal care [14,15]. Additionally, healthcare staff often struggle to recognise or appropriately manage difficult behaviours, a challenge frequently exacerbated by diagnostic overshadowing, where behaviours are attributed to the child’s disability rather than prompting further clinical assessment [15,16,17]. These issues are underpinned by limited staff training and negative attitudes toward disability [18].

Healthcare environments are typically designed for neurotypical populations (i.e., children without intellectual disability or neurodevelopmental disorders) and often fail to accommodate the distinct cognitive, sensory, and communication needs of children and young people with ID/DD. The absence of tailored support can lead to distress, substandard care, and increased risk of harm. The practice of reasonable adjustments, defined as adaptations to clinical processes, the environment, or care delivery, has emerged as a critical strategy for improving healthcare access, safety, and quality for this population [19,20]. Examples include adjusting communication (e.g., visual aids and social stories), tailoring environments (e.g., quiet waiting areas), changing how care is given (e.g., extended and flexible appointments), and providing sensory supports (e.g., sensory toys and musical instruments) [19,21,22,23,24].

There is emerging evidence that implementing reasonable adjustments can improve patient outcomes and experiences, particularly when supported by liaison roles, family partnerships, and clear protocols. In addition to improving individual encounters, reasonable adjustments facilitate better coordination of care by clarifying roles, improving information sharing and supporting continuity across providers and settings [19,21,22]. Even small adaptations, such as familiarisation visits, visual aids, and sensory accommodations, can reduce distress, enhance communication, and enable more coordinated care in paediatric and acute settings [25,26]. Despite this, implementation across services remains inconsistent, hindered by systemic discrimination, limited staff training, and unclear responsibility and processes [27,28]. Recent literature, therefore, emphasises the need to systematise reasonable adjustments through organisational frameworks and whole-of-hospital, interdisciplinary workforce training, reinforced by leadership and dedicated liaison roles [25,29].

In response, national guidelines have increasingly emphasised the importance of workforce development to build this capacity within healthcare systems. The Disability Royal Commission highlighted the need for strengthened health workforce training, calling for comprehensive education to equip health professionals with the necessary skills and knowledge to care for people with cognitive and intellectual disabilities [30]. Similarly, the National Roadmap for Improving the Health of People with Intellectual Disability outlines specific actions to enhance undergraduate, postgraduate, and continuing professional education in this area [31].

Existing research consistently highlights the importance of partnering with families to ensure safe, equitable, and responsive care for children and young people with ID/DD [14,32]. Parents often express a strong desire to work collaboratively with healthcare staff to advocate for the needs of their children and ensure optimal care [32]. Despite these advances and policy directions, there remains limited research on how care coordination practices are enacted in paediatric emergency departments for children and young people with ID/DD or how staff training interventions influence these practices. In particular, there is a paucity of qualitative research capturing both staff and caregiver perspectives on care coordination within acute care contexts.

The current study addresses this gap by examining care coordination through a secondary qualitative analysis of data collected during the evaluation of a novel staff training program, Motivated for Change. Delivered in the emergency department of a tertiary children’s hospital in New South Wales, Australia, the program was designed to improve care for children and young people with ID/DD. It aimed to do so by increasing healthcare staff knowledge, skills, and confidence in implementing reasonable adjustments (Ong et al., under review). The training incorporates motivational interviewing, flipped classroom methods, and process mapping and promotes the use of disability-specific resources, communication tools, and system-level processes such as flagging and referral pathways [15,33]. Care coordination was defined using the framework proposed by Schultz et al. [34]. The primary aim was to determine the presence of care coordination elements within the qualitative data from the pre- and post-intervention periods. The secondary aim was to explore whether the practices of care coordination were enhanced or influenced by the Motivated for Change program.

## 2. Materials and Methods

Setting: This study was conducted within the ED at The Children’s Hospital at Westmead (CHW), a tertiary paediatric hospital serving children and young people aged 0 to 16 years, primarily from the Western Sydney Local Health District (WSLHD), Sydney, New South Wales (NSW), Australia, as well as from broader regions across NSW and Pacific Island countries. Between 2023 and 2024, CHW recorded 64,789 ED presentations, including 379 Triage 1 (Resuscitation), 5499 Triage 2 (Emergency), 16,835 Triage 3 (Urgent), 41,215 Triage 4 (Semi-urgent), and 861 Triage 5 (Non-urgent).

Study Design: A qualitative, prospective cohort design was used to evaluate the practice of care coordination in the pre- and post-intervention periods of a training program, Motivated for Change, aimed at improving the reasonable adjustments that staff make for children and young people with ID/DD presenting to the ED. This study explored the experiences, knowledge, attitudes, and training needs of ED clinicians as well as the experiences and satisfaction of parents and caregivers during matched pre- and post-intervention periods. Hence, this study represents a secondary use of existing data, applying Schultz et al.’s framework and definitions of care coordination [34] to assess changes in practice.

The intervention involved three face-to-face training sessions (1 h each) delivered to ED staff between January and March 2023. Data were collected through direct observations of staff–patient interactions and semi-structured interviews with ED staff and caregivers of children and young people with ID/DD. During observations, some interactions may have involved staff who had not attended the training sessions due to factors such as training sessions not aligning with their rostered shifts. Participation was voluntary, and all data were deidentified prior to analysis.

Participants: Eligible participants included clinical staff employed in the ED at CHW during the study period and parents or caregivers of children with ID/DD.

ED staff: Email invitations were sent to ED staff to attend the Motivated for Change education sessions, and staff who were rostered during each session participated. Pre- and post-training surveys were completed by staff who attended all sessions to support self-reflection and evaluate the program. A total of 131 staff completed pre-training surveys, and 89 completed post-training surveys; however, these figures likely underestimate the number of staff who participated in the training, as survey completion was voluntary and not all attendees completed the survey. Staff who indicated interest during the post-training survey were invited to participate in optional online interviews. The results from the survey components are currently under review for a separate publication.

A total of 10 ED staff participated in interviews, including 4 at baseline and 6 post-intervention. At baseline, participants included 1 doctor, 1 nurse, and 2 child life therapists; all were female. Two participants were aged 30–39 years, 1 was aged 40–49 years, and 1 was aged ≥60 years. Two participants reported <5 years of clinical experience, 1 reported 5–10 years, and 1 reported >10 years. Two staff were permanently based in the ED, and 2 were rotating staff. Post-intervention, participants included 2 doctors, 3 nurses, and 1 child life therapist; all were female. One participant was aged 30–39 years, 4 were aged 40–49 years, and 1 was aged ≥60 years. One participant reported <5 years of clinical experience, and 5 reported >10 years. Four staff were permanently based in the ED, and 2 were rotating staff.

Parents and caregivers: Parents and caregivers were eligible to participate if they were caregivers for at least one child with ID/DD, had attended the CHW ED within the previous 6–12 months, and were available during the study period. Upon completion of the surveys, parents and caregivers were invited to participate in optional online interviews.

Five caregivers participated in interviews, including 2 at baseline and 3 post-intervention. At baseline, 2 parents (1 mother and 1 father) participated in a joint interview. Caregivers were aged 30–39 years and were caring for a child with a rare genetic condition. Post-intervention, 3 caregivers participated, including 2 mothers and 1 foster carer. Two caregivers were aged 30–39 years, and 1 was aged 60 years or older. Two caregivers identified as full-time carers, while 1 was in paid employment. Children represented a range of neurodevelopmental profiles, including autism spectrum disorder, intellectual disability, and a rare genetic condition. Caregiver demographic detail was intentionally limited due to the small sample size and the potential risk of participant identifiability.

Observations: Direct consent was not obtained from individual staff or caregivers present during observational periods, and no demographic information was collected for these participants.

Data Collection: Data were collected via observations and interviews conducted at two time points: pre-training and post-training. Baseline observations took place between November 2022 and January 2023, followed by baseline interviews between January and March 2023. Staff participated in the training between January and March 2023. Post-intervention observations were conducted between November 2023 and January 2024, and post-intervention interviews were conducted between December 2023 and March 2024.

**Figure 1:**
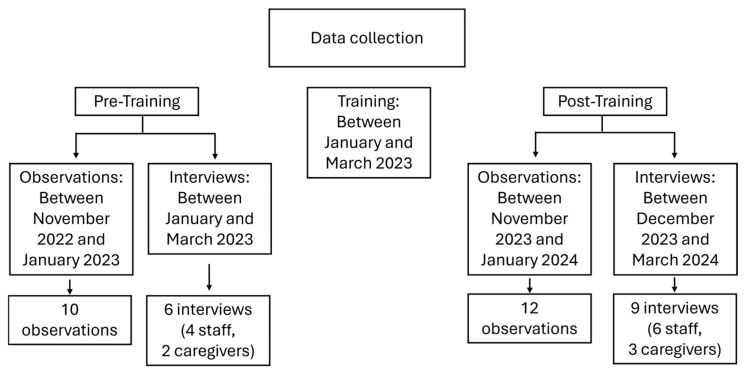
Timeline of data collection across pre- and post-intervention phases.

A total of 10 baseline and 12 post-intervention observations were conducted. Additionally, 6 baseline interviews (4 staff, 2 caregivers) and 9 post-intervention interviews (6 staff, 3 caregivers) were completed (see Figure 1).

For observations, the researchers informed the team leader in the allocated ED zone and identified patients with ID/DD. Signs were also displayed in the department to inform caregivers that research was being conducted. They then observed interactions between staff, the child or young person, and their caregivers. Following the observation, the researcher debriefed with the caregiver and invited them to participate in an interview. Information sheets were provided at this time.

Interviewers were trained using a guideline document developed by NO, a senior member of the research team who developed the intervention, and a specialist in developmental paediatrics. In staff interviews, clinicians were asked to reflect on recent experiences caring for children and young people with ID/DD (e.g., challenges, positive aspects, areas for improvement). Caregiver interviews focused on their most recent ED visit, with prompts exploring staff communication, child engagement, environmental factors, and perceived quality of care. Caregivers were also asked whether they had observed any differences compared to previous visits.

Data Analysis: Data were analysed using the framework method, a systematic and flexible approach particularly suitable for multidisciplinary health research teams [35]. The analysis was conducted by five members of the research team. The primary author (YK) was an advanced trainee in General Paediatrics. RB, Senior Speech Pathologist, and GT, a Clinical Nurse Consultant, both had expertise in supporting children and young people with ID/DD in hospital settings; AL, a Clinical Neuropsychology Registrar, and JM (PhD), a Research Officer, had experience in qualitative analysis. Consensus meetings were led by NO.

To enhance the rigour of this study, reflexive work was undertaken before the analysis commenced. Team members reflected on and discussed their professional backgrounds and potential influences on data interpretation, acknowledging how their clinical experiences might shape perspectives. Prior to the analysis, the research team met to discuss codes and came to a consensus decision of the themes. Bracketing was employed throughout the coding process to minimise bias. The team met regularly to review and refine the coding framework, reaching consensus through collaborative discussion. To ensure consistency, 30% of coded transcripts were randomly selected and reviewed by all team members. Data management and analysis were supported using NVivo software (version 14).

Ethics Approval: Ethics approval was granted from the Sydney Children’s Hospitals Network Human Research Ethics Committee (SCHN HREC 2022_ETH01993).

## 3. Results

The results are presented as themes and subthemes reflecting key domains of care coordination identified through qualitative analysis. To preserve narrative flow, illustrative quotes supporting coding and theme development are presented in Appendix B. These quotes broadly reflect the representation of themes in the dataset but are not intended to be proportional.

### 3.1. Communication Focused on Understanding the Child with ID/DD

#### 3.1.1. Implementation of the Top 5 Tile Enhances Interdisciplinary Communication

In the pre-intervention phase, a critical gap identified was the absence of a clear, accessible method to communicate the specific needs of children and young people with ID/DD among staff. The introduction of the Top 5 Tile, a digital alert embedded in an electronic ED triage system, addressed this by prominently displaying essential, child-specific adjustments within the child’s electronic medical health record. This initiative was designed to promote a shared understanding among clinicians about the unique needs of children and young people with ID/DD, both within the emergency department and across other departments. Staff were proactive in populating the Tile and took ownership of updating it as they learned more about each child and young person during the clinical encounter. The responsibility for maintaining the Top 5 Tile was distributed across disciplines, including junior and senior nurses, junior and senior doctors, and child life therapists. Caregivers reported improved experiences and attributed better care to the consistent transfer of child-specific knowledge facilitated by the Top 5 Tile. While time constraints and limited training were cited as barriers, active peer-to-peer teaching and staff enthusiasm helped embed this tool into the daily workflow. Notably, the process also encouraged caregivers to reflect more deeply on their child’s strengths and support needs.

#### 3.1.2. Building Trust with Caregivers Through Child-Centred Communication

Effective communication, from the caregiver’s perspective, involved three key elements: asking child-focused questions, listening to caregiver concerns, and sharing decision-making with parents and caregivers. Prior to the intervention, while some good communication practices were observed, they were inconsistent and predominantly focused on medical issues rather than psychosocial needs. Following the training, staff more consistently asked about past hospital experiences, sensory triggers, and coregulation strategies, such as caregiver presence and guided distraction.

This shift toward holistic inquiry improved caregiver satisfaction and enhanced collaborative care. Instances of poor child-centred communication were infrequently reported and were predominantly described by a single caregiver, where parental expertise was not actively acknowledged.

#### 3.1.3. Child-Friendly Communication Facilitates Engagement

Baseline data showed a lack of engagement with minimally verbal children and young people and a limited understanding of how to assess communication abilities. While some staff used developmentally appropriate language and behaviours, such as playing games or child-friendly metaphors, these practices were inconsistent.

Following the intervention, there was a marked improvement in staff efforts to assess and adapt communication to each child and young person’s needs. Staff more consistently involved children and young people in procedures and assessments, acknowledged individual needs, and tailored communication strategies to their developmental level. This shift is likely to reflect increased staff confidence and awareness prompted by targeted training. These interactions not only put children and young people at ease but also demonstrated respect for their personhood, strengthening family trust in the clinical team.

### 3.2. Individualised Assessment Guides Tailored Adjustments

#### 3.2.1. Recognising Unique Needs Through Targeted Inquiry

Post-intervention, staff demonstrated greater awareness of the need to tailor care to each child and young person’s sensory, communication, and developmental profile. Staff more frequently inquired about triggers and calming strategies and adjusted the physical environments accordingly—for example, choosing between open bays and single rooms based on individual needs. In contrast, baseline data showed that such assessments were often overlooked due to time pressures and task-oriented practices. Staff also acknowledged challenges in identifying ID/DD, particularly in the absence of a prior diagnosis, and highlighted a lack of confidence and tools as barriers to effective assessment.

#### 3.2.2. Caregivers as Experts in Their Child’s Needs

Both before and after the intervention, staff recognised caregivers’ expertise and involved them in decisions about environmental setup, communication strategies, and procedural planning. However, post-intervention data indicated a more structured and proactive approach in eliciting caregiver input. Staff more explicitly acknowledged the value of caregiver insights in managing complex needs, which contributed to stronger partnerships and more effective care delivery.

### 3.3. The Expanding Role of Child Life Therapists (CLTs)

#### 3.3.1. Advocacy for Children and Young People and Families

Child life therapists were consistently observed to advocate for both children and young people with ID/DD and their caregivers, ensuring that adjustments were implemented and voicing concerns when care plans were misaligned with the child’s needs. Post-intervention data underscored their increased visibility and influence, particularly through their role in completing and updating the Top 5 Tile.

#### 3.3.2. Educating Staff and Caregivers

CLTs served as key educators, modelling how to understand and effectively support communication, teaching disability-sensitive practices, and helping staff to assess and support children and young people with complex needs. They also empowered caregivers by facilitating reflection on their child’s needs and reinforcing strategies that work well.

#### 3.3.3. Direct Support for the Child

CLTs contributed significantly to procedural success and emotional regulation by engaging in preparatory work, distraction techniques, and individualised desensitisation strategies. Their support enabled children and young people to undergo procedures with reduced distress and better cooperation, which led to completion with less restraint, improving the patient experience and clinical outcomes.

#### 3.3.4. Supporting Parents Through Trust and Communication

CLTs played a pivotal role in building rapport with caregivers, particularly those initially reluctant to engage. Their thoughtful, developmentally informed questioning often created a safe space for disclosure and helped bridge communication gaps between caregivers and medical teams.

#### 3.3.5. Increased Integration Post-Intervention

Post-intervention data indicated a substantial increase in referrals to and appreciation of CLTs, contrasting with pre-intervention reports of limited awareness and inconsistent engagement with the role.

### 3.4. Developing Individualised Care Plans

#### 3.4.1. Collaborative Planning with Families

Staff increasingly engaged children, young people and caregivers in codeveloping care plans that accounted for regulation supports, medication preferences, and communication strategies. This collaborative approach fostered trust and improved the suitability and success of interventions.

#### 3.4.2. Maintaining Calm and Reducing Sensory Overload

Post-intervention practices showed a significant rise in the use of low-sensory environments, distraction techniques, and preventative behavioural strategies. These included fast-tracking children and young people through care pathways and planning for escalation, reflecting a shift toward a more anticipatory, child-centred model of care.

#### 3.4.3. Addressing Barriers to Personalised Care

While improvements were notable, barriers such as time constraints, workforce pressures, and inconsistent planning continued to challenge the delivery of optimally individualised care.

#### 3.4.4. Procedural Planning and Preparation

Compared to the baseline, staff post-intervention were more deliberate in preparing children and young people for procedures, both emotionally and physically. The use of numbing agents, procedural rehearsal, and distraction was more common. In the post-intervention data, one particularly effective communication strategy highlighted was the One Voice approach, in which only one designated person spoke to the child or young person throughout the procedure, minimising unnecessary stimuli. However, a single observed instance of unnecessary physical restraint highlighted the need for ongoing emphasis on trauma-informed practices.

### 3.5. Transitions of Care from the ED

In the baseline data, information about a child’s disability was sometimes omitted during nursing handovers from the ED to inpatient wards. Comparable data were not captured post-intervention, limiting direct comparison. However, handovers between the ED and radiology department demonstrated improvement following the introduction of the Top 5 Tile.

### 3.6. Impact of the Motivated for Change Program

Staff widely described the training program as transformative, with reported benefits including clearer frameworks for understanding and supporting ID/DD, improved teamwork, and a shared language for discussing behavioural escalation. The education on escalation curves, structured communication, and disability awareness equipped staff with practical tools to navigate complex situations more calmly and effectively. This increased the confidence of staff and helped in their communication with parents and caregivers.

### 3.7. Alignment of Themes with the Care Coordination Framework by Schultz et al. [34]

The measurement framework described by Schultz et al. [34] includes 14 domains (see Table 1), and themes from this study were mapped to seven domains (see Table 2). The most frequent alignment occurred with Communicate, Assess needs and goals, Create a proactive plan of care, and Align resources with patient and population needs. Codes were also identified for Establish accountability or negotiate responsibility and Teamwork focused on coordination, though these were less frequent and did not form distinct themes. No data were coded to the remaining domains, suggesting they were not represented in the dataset.

## 4. Discussion

The findings suggest that the Motivated for Change program had a meaningful impact on enhancing care coordination in a busy, high-pressure setting like the ED. The introduction of structured tools such as the Top 5 Tile helped overcome communication barriers by ensuring that individual, child-specific needs were visible, shared, and acted upon. This facilitated a more unified and proactive approach to care.

Clinicians demonstrated a shift toward more holistic, child- and family-centred communication, indicating a growing awareness of the psychosocial and behavioural dimensions of disability, not just the medical. The increased involvement of caregivers as experts in their child’s care represents a positive move toward shared decision-making, aligning with best-practice principles of family-centred care.

The strengthened role of child life therapists further demonstrates that embedding specialised roles within the ED can improve the quality of care, particularly for children and young people with complex needs. Their ability to provide advocacy, education, emotional support, and procedural preparation filled critical gaps in care delivery and trust-building with families.

Despite systemic challenges like time constraints and limited staffing, staff embraced the program and perceived it as transformative. This reflects both the feasibility and acceptability of implementing targeted training in real-world clinical settings. Importantly, the intervention not only increased staff confidence but also resulted in observable changes in practice, suggesting that improving care coordination is achievable with the right support and tools. Caregivers’ enhanced experiences suggest that even small changes in language, listening, and collaboration have a meaningful impact.

These results collectively indicate that tailored, disability-specific education and systems-based tools can lead to more individualised, coordinated, and compassionate care for children and young people with ID/DD in acute settings.

**Interpretation of alignment with the Care Coordination Framework**

The strong representation of Communicate, Assess needs and goals, Create a proactive plan of care, and Align resources with patient and population needs highlights the central role of information exchange between staff, relationship-building with families, early needs assessment, and resource coordination in the ED. These activities align with the fast-paced nature of emergency care, where time-sensitive decision-making and collaboration across team members are essential.

The less frequent presence of Establish accountability or negotiate responsibility, Facilitate transitions, and Teamwork focused on coordination reflects structural and operational features of the ED. The responsibility for ongoing coordination and transitions of care often shifts to inpatient teams or community-based services following ED discharge, reducing the scope for these domains to be fully enacted in the ED setting. Role negotiation occurred at two levels: among team members and between staff and parents or caregivers. Although data relating to negotiation with caregivers were limited, existing literature suggests that clarity around roles and responsibilities is important for building trust and supporting parents to feel confident in allowing staff to assume care of their child [36].

Domains absent from the data represent activities less relevant in the ED, such as long-term follow-up planning. Their absence does not necessarily indicate deficiencies in care but rather reflects the framework’s broader applicability across settings beyond acute care. Overall, these findings suggest that, while Schultz et al.’s framework [34] applies to ED-based care coordination, certain domains are more prominent and context-dependent, underscoring the need to tailor coordination strategies to the specific demands of the healthcare environment.

Existing literature highlights the significant challenges faced by children and young people with ID/DD in healthcare systems [14,15]. Parents frequently report inconsistent recognition of disability, limited reasonable adjustments, delayed triage, and poor communication [37]. These findings mirror our baseline data, where gaps were observed in direct communication with the child and young person, partnerships with families, and implementation of reasonable adjustments.

Our findings also align with prior research emphasising the importance of targeted staff training in improving care for children and young people with ID/DD [25,26]. Consistent with Moloney et al. [25], liaison and advocacy roles such as child life therapists played a pivotal role in facilitating adjustments, improving staff capacity, and fostering a culture of inclusion within the ED.

Due to the paucity of studies in examining care coordination in the ED setting for children and young people with ID/DD, this study contributes to this sparse evidence base by demonstrating how care coordination translates into tangible practices that are both meaningful to families and feasible for staff. Our post-intervention findings illustrate that structured tools such as the Top 5 Tile, proactive caregiver engagement, and integrated liaison roles like child life therapists can operationalise care coordination in ways that improve continuity of information, staff confidence, and the family’s sense of partnership in care. These real-world applications extend beyond theoretical frameworks, illustrating what coordinated care “looks like” in practice.

**Strengths and Limitations**

This study used a qualitative design, combining observations and interviews to enable triangulation and capture both practice and perspectives. Inclusion of staff and caregiver voices enriched the data and ensured that the findings reflected both sides of the care experience. However, the voices of children and young people were not included.

The pre- and post-design allowed us to assess changes over time, capturing the real-world impact of the intervention. However, being a single-site tertiary ED study limits generalisability, and recruitment of caregivers was challenging. Only a small number of parents were able to participate, and only limited demographic information was included to maintain participant confidentiality. While the sample was small, caregiver perspectives were triangulated with observational data to strengthen the findings.

Some interviews were conducted by intervention team members; however, potential bias was mitigated by having the majority of interviews conducted and coded by non-intervention researchers (including the first author, RB, AL, and JM), with high coder agreement. While the team’s prior clinical experience helped with the interpretation of the data, it also posed a risk of bias. This was addressed through reflexive practices such as bracketing and regular debriefs to ensure participant voices were authentically represented.

Limited data were collected regarding transitions of care from the ED, preventing evaluation of whether the intervention improved practices in this domain. Sustainability of the program beyond the study period was not formally evaluated; however, the program continues to be delivered by the internal team at regular intervals, including during staff changeovers, at least every 3 months for junior doctors.

**Implications**

The Motivated for Change program demonstrated that targeted disability-focused training, combined with practical system-level tools and dedicated roles such as child life therapists, can support more equitable, coordinated, and family-centred care in the ED. Embedding these approaches into routine practice aligns with national priorities under the Disability Royal Commission and the National Roadmap for Improving the Health of People with Intellectual Disability [30,31].

**Future Directions**

Future work should explore the factors that support sustainability of training programs such as Motivated for Change, including the development of local champions to maintain ongoing delivery and engagement. Research could also examine adaptation and scaling across other hospital settings and departments. While this study was qualitative, future studies could integrate quantitative measures, including operational ED data and patient outcomes, to complement qualitative findings. Incorporating the perspectives of children and young people would further strengthen understanding of care experiences and guide program refinement.

## 5. Conclusions

This study found that the Motivated for Change program improved care coordination for children and young people with ID/DD in the emergency department. Key outcomes included clearer identification and communication of individual needs through the Top 5 Tile, more consistent child- and caregiver-centred communication, and greater use of anticipatory planning and environmental adjustments. These changes contributed to safer, more coordinated care and improved family experiences. Ongoing investment in workforce training, inclusive systems, and strong caregiver partnerships is essential to sustain and expand these practices.

## Figures and Tables

**Table 1:** Measurement framework definitions (adapted from Schultz et al.) [34] (detailed framework in Appendix A).

Framework Element	Definition
Domains: Coordination activities	
Establish accountability or negotiate responsibility	Clearly define who is responsible for each aspect of a patient’s care, including what they are accountable for and when responsibility shifts to another person or team.
Communicate: - Interpersonal communication - Information transfer	Ensure effective knowledge sharing among all involved in a patient’s care. For evaluation, communication is categorised as interpersonal or information transfer.
Facilitate transitions: - Across settings - As coordination needs change	Efforts to support care transitions. These include transitions across settings (e.g., hospital to outpatient care) and transitions as coordination needs change (e.g., acute to chronic care).
Assess needs and goals	Identify the patient’s care and coordination needs by assessing their physical and psychological health, functional status, medical history, treatment plans, and support service requirements.
Create a proactive plan of care	Create and maintain a care plan collaboratively with the patient, family, and healthcare team.
Monitor, follow-up and respond to change	Together with the patient and family, regularly review progress toward care and coordination goals.
Support self-management goals	Provide education and support that match the patient’s capacity and preferences for involvement in their care.
Link to community resources	Inform patients about available community resources and, if needed, help coordinate access to them.
Align resources with patient and population needs	Assess and respond to both individual and population-level care needs by aligning healthcare resources accordingly.
Domains: Broad approaches potentially related to care coordination	
Teamwork focused on coordination	Integrate healthcare professionals involved in a patient’s care into a cohesive and collaborative unit, ensuring they function together effectively to meet the patient’s needs.
Healthcare home	A patient’s chosen usual source of care coordinates care according to their needs and preferences.
Care management	A process that helps patients and their support systems manage medical, social, and mental health conditions more effectively.
Medication management	Medication reconciliation ensures accurate medication lists during care transitions to prevent errors.
Health information technology (IT)-enabled coordination	Tools like electronic medical records, patient portals, or databases support communication and continuity of care between healthcare providers and over time.

**Table 2:** Alignment of Schultz et al. measurement domains with findings from this study [34].

Schultz et al. Care Coordination Domains	Themes from This Study
Communicate—Interpersonal communication Communicate—Information transfer Health IT-enabled coordination	3.1. Communication focused on understanding the child with ID/DD
Assess needs and goals	3.2. Individualised assessment guides tailored adjustments
Align resources with patient and population needs	3.3. The expanding role of child life therapists (CLTs) 3.4. Developing individualised care plans
Create a proactive plan of care	3.4. Developing individualised care plans
Facilitate transitions	3.5. Transitions of care from the ED
Monitor, follow-up, and respond to change	3.6. Impact of the Motivated for Change program

## Data Availability

The data that support the findings of this study are available on request from the corresponding author. The data are not publicly available due to privacy and ethical reasons.

## Appendix A

**Table A1:** Detailed summary of the care coordination measurement framework (adapted from Schultz et al., 2013) [34].

Framework Element	Definition
Domains: Coordination activities	
Establish accountability or negotiate responsibility	Clearly define who is responsible for each aspect of a patient’s care, including what they are accountable for and when responsibility shifts to another person or team. The designated individual, team, or organisation must be able to answer for any failures related to their part in the care process.
Communicate: Interpersonal communication Information transfer	Ensure effective knowledge sharing among all involved in a patient’s care. For evaluation, communication is categorised as interpersonal (two-way, direct interaction) or information transfer (one-way sharing of data without direct contact), even though both often occur together in practice.
Facilitate transitions: Across settings As coordination needs change	Efforts to support care transitions focus on times when responsibility or information is transferred between providers or maintained over time. These include transitions across settings, such as from hospital to outpatient care, and transitions as coordination needs change, like moving from paediatric to adult care or from acute to chronic care management. All transition measures fall within one or both of these categories.
Assess needs and goals	Identify the patient’s care and coordination needs by assessing their physical, emotional, and psychological health, functional status, medical history, treatment plans, self-management abilities, and support service requirements.
Create a proactive plan of care	Create and maintain a care plan collaboratively with the patient, family, and healthcare team. The plan should address ongoing needs, identify coordination gaps, and set goals for both the patient and providers. It should also anticipate routine needs and track progress over time.
Monitor, follow-up and respond to change	Together with the patient and family, regularly review progress toward care and coordination goals. Monitor for successes and setbacks, update the care plan as needed based on new information or circumstances, and ensure appropriate follow-up care is provided.
Support self-management goals	Provide education and support that match the patient’s capacity and preferences for involvement in their care. This includes information, training, or coaching to help patients and caregivers understand and manage self-care tasks, navigate transitions, and build confidence and positive behaviour change.
Link to community resources	Inform patients about available community resources and, if needed, help coordinate access to them. These may include financial aid, social services, educational programs, support groups, or wellness services that support the patient’s health and care goals.
Align resources with patient and population needs	Assess and respond to both individual and population-level care needs by aligning healthcare resources accordingly. For populations, this may involve creating system-level strategies, such as multidisciplinary care teams. For individual patients, this includes evaluating whether they would benefit from these approaches to enhance coordination and streamline care.
Domains: Broad approaches potentially related to care coordination	
Teamwork focused on coordination	Integrate separate healthcare professionals, teams, or organisations involved in a patient’s care into a cohesive and collaborative unit, ensuring they function together effectively to meet the patient’s needs.
Healthcare home	A patient-chosen usual source of care acts as the central hub for coordinating all aspects of care based on the patient’s needs and preferences. Known as a medical home or patient-centred medical home, this model ensures coordinated, accessible, and comprehensive care through a team-based, system-driven approach focused on quality and safety.
Care management	A process that helps patients and their support systems manage medical, social, and mental health conditions more effectively. This includes approaches like case management and disease management.
Medication management	Medication reconciliation ensures accurate medication lists during care transitions to prevent errors, involving review across settings and clear communication with patients and providers.
Health information technology (IT)-enabled coordination	Tools like electronic medical records, patient portals, or databases support communication and continuity of care between healthcare providers and over time.

## Appendix B

**Table A2:** Selection of illustrative quotes supporting the coding and theme development.

**Theme 1: Communication Focused on Understanding the Child with ID/DD**			
	Subtheme 1: Implementation of the “Top 5 Tile” Enhances Interdisciplinary Communication	Subtheme 2: Building Trust Through Child-Centred Communication	Subtheme 3: Child-Friendly Communication Facilitates Engagement
**Baseline**	“It’s okay when they are already diagnosed, but probably we lack tools to recognise them in [the] emergency [department].” “There was no way of flagging in the system but thought this would be a good idea and maybe a brain icon with question marks as an identifier.”	“Observed this type of interaction with junior to senior medical staff handover. It had a very diagnostic focus and treatment care path [ways].” “Mum stated that no one is listening to her concerns or willing to help her.” “Doctor was using good questions to try and establish what was different with him and trying to find ways that would assist with care management.”	“You talk more to the carer than you do to the child. I’ve gotta [got to] reflect upon not engaging much time with the actual child.” “Observed doctor communication with parents, didn’t interact with child.” “The nurse then proceeded to feel for a pulse and used round and round the garden as a way to get a hold of his wrist whilst the carer was holding his other hand.”
**Post-intervention**	“It’s simple things like just asking the parents, What does their child like? And the things like that have made quite a big impact. The Top 5 Tile has been really well received.” “So, I referred to the puzzle piece [Top 5 Tile] and that gave me the stuff that I needed to look out for. I didn’t have to ask mum again. I had a rough guide on how to best look after this patient.” “This came from the Top 5 Tile, that this person liked music, and that was a good way of calming him.”	“[Before the training], we would never have asked the mother so many questions. The child would have been in an open area. Then the child would have escalated and then we would have had to use, you know, verbal de-escalation, chemical solution, all sorts of things. So I think we, the planning that has gone now into thinking about what could go wrong for these kids or what do we need to do for these kids to be quiet. Calm is what I think the staff are thinking quite actively.” “Whatever the parents thought was good for a distraction, and we did it at his level and in his time and let him check out everything because they are paying attention to what’s around them, even if they’re not making eye contact with you and responding to the words.”	“He [triage nurse] got down to her level at some points as well, which was good, like getting down to the child’s eye level is really good.” “So that’s three different staff and they all did the same thing. They were all making sure that they had eye contact, talking to [my child], and then they proceeded to do what they were doing.” “She [nurse] explained what she was going to do and showed the child the equipment.” “I laid on the ground with him and I got the two or three different bandages and rubbed them against his face.”
**Theme 2: Individualised assessment guides tailored adjustments**			
	Subtheme 1: Recognising unique needs through targeted inquiry	Subtheme 2: Caregivers as experts in their child’s needs	Subtheme 3: Assessing the child’s communication abilities
**Baseline**	“Sometimes [nurses] will say to me [child life therapist], they’ve got a child with autism spectrum disorder (ASD). ‘How do I support them?’ Sometimes it can be a bit overwhelming for them or they might feel a little out of their element in how to support children who have intellectual disabilities, because there’s no black and white answer. There’s no step-by-step directions of, first you do this, then you do this.” “I find we do their nursing care, but we don’t have the time to spend with them. So these poor kids are left on their own. We’re just so time poor. We’re so task oriented. That’s our biggest problem.”	“Mum then explained to nurse her ongoing concerns regarding jerky movements and need for neuro review and that he was damaged by the haloperidol. [Observer] could see over the interaction with health staff that mum was always trying to get someone to listen to her about her concerns but even though they acknowledged her, no one was delving into the issues.”	“And because we [ED staff] don’t know how they communicate. I suppose you don’t really kind of take into account what they do understand.” “Unclear if doctor had checked regarding communication levels earlier, but [the doctor] didn’t ask when doing [a] throat check. [The doctor] mostly spoke at parents, rather than involving child in conversation.”
**Post-intervention**	“She [carer] stated that staff could see straight away her child would need support.” “We also know that these kids are higher risk kids so they can come with sepsis and it’s so easy to miss, you know, serious infections, sepsis, all sorts of things. So, this group is very challenging.” “Now with the Motivated for Change program, where we’re a bit more conscious of asking about what the triggers and what their issues are.”	“I generally tend to ask the parents what will work for them in this kind of environment and what we can do to help make that better for them, which is very difficult sometimes when it’s like the middle of winter, it is very busy here trying to do as best we can for them. [It] can be a little bit hard because some kids, they need a separate space.”	“She [child life therapist] just observed a lot before interacting with her.” “You actually approach him a bit more differently because you know that probably you need to just explain things more to him than another child. So, you definitely do get the sense that you have to approach that child differently after that little bit to check conversations.”
**Theme 3: The expanding role of child life therapists (CLTs)**			
	Subtheme 1: Advocacy for children and young people and families	Subtheme 2: Educating staff and caregivers	Subtheme 3: Direct support for the child
**Baseline**	“I [CLT] did have a really positive experience where I had to advocate for a child with a member of staff, one of the doctors, who was not very appreciative of my advocacy and had a word with me about it afterwards, that was overheard by the nurses. I said, ‘I hear what you’re saying and I recognise that must be frustrating for you that my advocacy is making you feel a certain way. I apologise for that, and if circumstances had been different, I would have approached the situation differently, but unfortunately, this situation was what it is and this is my job.’” “I [CLT] was like, okay, I’m going to talk to the team. I felt like I was pretty much advocating, sharing her voice, asking the team, ‘Where’s the medication? Mum is really concerned about that. Mum is concerned about this room. Can we move him to a different room?’”	“It’s a department that has very high staff turnover. It’s a tertiary hospital, so doctors rotate. A huge part of my [CLT] time over there is educating and advocating for my role. You’re constantly re-educating nurses, re-educating doctors.” “Sometimes they’ll say to me, they’ve got a child with ASD, ‘How do I support them?’ ‘I’m like, they all present so differently. They’re so unique that you can’t, I can’t give you a magic wand answer. You need to talk to the parents. You need to know the right questions to ask. Ask them. Have they had any prior experiences? What did they look like? What do they find challenging about the hospital? What do they find comfortable? What does it look like when they’re emotionally distressed? What kind of behaviours do they seek certain sensory input, avoid certain sensory input?’”	“When we [CLTs] play with children, we consider play whether they’re big [or small], so there’s value in the play and [fostering] hopes. And it helps them to cope, and it also helps them to be laying [in] the presence of someone who is emotionally safe as well. So that’s the value of it.” “I [CLT] would probably engage them in more therapeutic play experiences to help them to normalise the hospital [experience].” “I [CLT] supported with some pain management strategies.”
**Post-intervention**	“The medical team wanted to go one way and the child life therapist is like, that’s not going to work for [this patient].” “I was able to advocate for things like making sure the numbing cream was on for a solid hour as opposed to 30 min.”	No quotes relevant to this subtheme were identified in the post-intervention dataset.	“They [medical team] got the child life therapist in. She [CLT] spent a lot of time with this patient.”
	Subtheme 4: Supporting parents through trust and communication	Subtheme 5: Increased integration post-intervention	
**Baseline**	“His mum was very agitated. She was very protective. The moment I [CLT] walked in the room, I knew there was repairing that needed to happen. As I engaged with mum, I started asking her very developmentally based questions. I saw the moment the lightbulb went off, and she realised, oh, okay. She’s like, ‘Okay, he hates lights. He likes vibrations. He finds too much auditory input overwhelming. When he becomes emotionally dysregulated, this is what it looks like. There are the things I need from you guys to help him be emotionally regulated.’” “I’ve [CLT] worked to hope that they could trust me and do the build a relationship and then based on that I would provide [care].”	“It’s been hard work. It all comes down to different staff, their training, level of experience, how often we’ve been able to work with them, the relationships we have with them and their perception of what child life therapy is. When I first went over there, I had a consultant who was like, ‘Oh, it’s the playdough lady. Can you fill up all the playdough? This child needs playdough. That child needs bubbles.’” “We [CLT] learnt we had to be physically there, have our feet on the ground and we needed to be seen for them [ED doctors and nurses] to use us.”	
**Post-intervention**	No quotes relevant to this subtheme were identified in the post-intervention dataset.	“We [ED staff] definitely involve her [CLT] more often after the training to help.” “It was then decided that child would need a blood test and possibly a cannula as [he was] not drinking. The nurse stated at this point, she was able to contact and flag the child with [the child life therapist] who started working on desensitising him to torniquet and procedure and working on distraction techniques.”	
**Theme 4: Developing individualised care plans**			
	Subtheme 1: Collaborative planning with families	Subtheme 2: Maintaining calm and reducing sensory overload	
**Baseline**	“They [ED staff] needed to cannulate him. So long story short, he [patient] was too fearful, so he ran away. He absconded from the bedside. Then I [CLT] told the nurses the plan. ‘Let’s try this plan.’ Let’s and the care is also changed over and the carers had a relationship with him as well. But he got a connection with me. So I facilitated with the carer. And I told the carer, educated the care and what to do and how to respond and what to say and came in and modelled as appropriately, just to avoid crowding him.” “Doctor asked about current medications which [were] melatonin and clonidine. She then discussed about giving him an ondansetron wafer which dissolves in the mouth. She asked if he would eat an ice block and that they would try to put wafer on this.”	“[Observer] attended the Acute Care Area and observed a boy recently brought in on a background of ASD, ADHD, intellectual disability, emotional dysregulation and again unwell. He [was] accompanied by his mother and a NDIS support worker. [He was] placed in a single room near the nurses station.”	
**Post-intervention**	“[Observer] liked that [the] doctor was listening and acknowledging mother’s concerns. Also that these concerns were investigated by team and something was done about it. I felt mum was being heard and she felt comfortable and had a tear in her eye.” “Whatever the parents thought was good for a distraction, and we did it at his level and in his time and let him check out everything because they are paying attention to what’s around them, even if they’re not making eye contact with you and responding to the words.”	“She [nurse] also stated that she has seen a big change with the medical staff due to [senior doctor’s] input and finds them more accommodating to see the children and use of single rooms to assist with sensory overload.” “I [triage nurse] also tend to sort of go inside and if I have a fairly good working rapport with most of the consultants and sort of say, ‘Oh look, I’ve got this kid and if I can find them a single room, do you think we can [use the room]?’” “It was quiet and lights were dimmed.”	
	Subtheme 3: Addressing barriers to personalised care	Subtheme 4: Procedural planning and preparation	
**Baseline**	No quotes relevant to this subtheme were identified in the baseline dataset.	“Sometimes staff might feel that it’s easier and safer to physically restrain the child to get the job done and worry about the consequences later, rather than investing the extra time it might take to page allied health, get the right planning in place, having those conversations with the family, knowing what questions to ask, and investing time there.” “Sometimes you can only make the best choice that you can make in the current context that you have. A huge thing, I think one of the huge constraints in ED is time and staffing. We don’t, there’s often not enough time. The pressure to get the patients in and out of ED in under four hours means that staff feel the time pressure to get things done and sometimes leads to decisions that might not be best practice.”	
**Post-intervention**	“Interviewer: What do you think was the issue before you came on that made it quite difficult and challenging? Staff: The brightness of the department. Maybe they didn’t have enough time to spend with him and knowledge too that not, you know, realising this is not [going to] work for this particular child.”	“After the training, it just helped us to ask ourselves, ‘Is [the procedure] really needed?’ like sometimes like when a practitioner asks for [a] blood test. Is it really needed that we need that blood test? [Would it] change our management? Because it’s always [distressing] and difficult.” “We had to administer nitrous oxide to a patient who had an intellectual disability [and] autism and it was an examination of his eye. That’s what it was. And he is like, didn’t want the drops to be put in. So the team just said nitrous oxide would be the best thing to do. And so it was really like the thing that I really appreciated the most out of this was there was a lot of time put into preparing. It was a chaotic day, very busy. But we identified that this was a patient who would really benefit from taking the time to make sure that we did this properly because we didn’t want to make it a bad experience for him because we understood that could potentially have a really bad flow on effects. This was a child would have to come to hospital again most likely.”	
**Theme 5: Transitions of care from the ED**			
**Baseline**	“When people are rushed and [they] just want to go home. I think when people get stressed when they are rushed and they just want to give the basic kind of handover. I think that’s where it [history of intellectual disability] can be missed.”		
**Post-intervention**	“Nursing staff then commented on how they felt that the program has taught them a lot and feel that the Top 5 [Tile] is easy to complete and has made them more aware of finding out about the child as it does make a difference to care. They also mentioned that they noted that Top 5 Tile [was used] in [the] radiology [department] and that they noted that radiology will ring to find out more about the child to assist with preparation.”		
**Theme 6: Impact of the Motivated for Change program**			
**Post-intervention**	“She [carer] stated that she could feel the change in staff’s interest and care she has received.” “[Prior to the program], I was basing all of my care on a story that someone told me where there was a burns patient who was going in and out of consciousness. The nurse would say ‘I would still talk to them as if they were still in there and they could understand everything that I was saying.’ So that was kind of how I approached my care when it came to looking after children with intellectual disability.” “I guess before the training, maybe I was always a bit intimidated when like a patient with a disability presents to the emergency [department]. Partly because you never know what to expect and you don’t know whether you can manage if things go wrong.” “There’s now an algorithm that outlines those reasonable adjustments. The way that we can involve the parents is a bit more specific rather than just, you know, ‘what can we do? What do you think can help?’ We can actually ask specific questions that will help to guide our care. And we can put that into a management plan.” “[This] Project has been a real game changer for us and I think it’s been really helpful as the staff love this project because we still see a lot of kids with autism and intellectual disabilities.”		
